# Dental Implants Placed in Grafted and Non-Grafted Sites: A Systematic Review

**DOI:** 10.3290/j.ohpd.b5828032

**Published:** 2024-11-18

**Authors:** Seymur Gurbanov, Philipp Plugmann

**Affiliations:** a Seymur Gurbanov Dentist, Dental Office of Oral Surgery and Implantology, Bergisch Gladbach, Germany. Privat University in Fürstentum Liechtenstein (UFL), Triesen, Liechtenstein. Study design, literature search, wrote and reviewed the manuscript.; b Philipp Plugmann Professor of Interdisciplinary Periodontology and Prevention, SRH University of Applied Health Sciences, Leverkusen, Germany. Supervision of the study, wrote and reviewed the manuscript.

**Keywords:** bone graft, dental implant, guided bone regeneration, survival, 10 years

## Abstract

**Purpose:**

There is a scarcity of data regarding the long-term follow-up of dental implants placed in grafted or non-grafted sites. The aim was to systematically review clinical studies which, compared the implant survival rate (ISR) after at least 10 years for dental implants placed in grafted and non-grafted sites.

**Materials and Methods:**

The focused question addressed was: ‘Is there a difference in the ISR of dental implants placed in grafted and non-grafted sites for at least a decade?’ The inclusion criteria were: (a) clinical studies, (b) studies on patients who had undergone dental implant therapy, (c) studies with at least 10 years follow-up, and (d) studies that compared the clinical and radiographic status around implants placed in grafted and non-grafted sites. Indexed databases (PubMed/Medline, Scopus, EMBASE, OVID, ISI Web of Knowledge, and Google Scholar) were searched without time and language restrictions up to and including December 2020 using different keywords. The literature search was performed in accordance with the Preferred Reporting Items for Systematic Reviews and Meta-Analyses (PRISMA) guidelines. The risk of bias was assessed, and the pattern of the present systematic review was customised to summarise pertinent information.

**Results:**

The initial search yielded 412 studies through electronic database searching. An additional 30 studies were identified through manual searching of full texts of studies. In total, three studies were included and processed for data extraction. In these studies, the number of participants ranged between 34 and 96 individuals. The mean age ranged between 47.2 and 67.6 years. The reported ISR ranged between 91.6% and 100%. All studies had a low risk of bias. Due to the high heterogeneity among the studies included, a meta-analysis could not be done. Prior sample-size estimation was done in none of the studies.

**Conclusion:**

Dental implants placed in grafted and non-grafted sites demonstrate similar ISR for at least a decade. However, further well-designed and power-adjusted studies are needed.

Dental implants have revolutionised clinical dentistry and related research providing a reliable solution for the replacement of missing teeth.^1,11,34,35^ Studies^2,15,30,44^ have shown that dental implants demonstrate success and survival rates of up to 100%; and can remain functionally stable in medically compromised and systemically healthy individuals.^23,25,26^ The long-term success of dental implants is influenced by various factors, including the achievement of primary stability, and quality and quantity of the alveolar bone at the implant site.^3,16^ When the bone volume is insufficient, bone grafting procedures are often employed to enhance the site and improve implant stability and survival rates.

Bone grafting techniques are widely used to address alveolar bone deficiencies resulting from various factors including periodontal disease, and/or immediate implant placement after tooth extraction.^8,12,22,39^ Surgical interventions utilised to induce new bone formation (NBF) in osseous defects are collectively known as ‘guided bone regeneration (GBR)’.^22,37,43^ Several studies^7,13,32,41,42^ with follow-up durations ranging between 12 and 72 months have shown that dental implants, when placed in grafted sites, demonstrate osseointegration and clinical stability which is comparable to implants placed in non-grafted sites. It is however pertinent to mention that clinical factors such as systemic health, smoking status, and oral hygiene maintenance can influence the clinical and radiographic status of dental implants placed in grafted and non-grafted in the long term (at least a decade after placement). Moreover, long-term studies provide more valuable insights into the durability and predictability of implants placed in grafted and non-grafted sites, contributing to evidence-based clinical decision-making. The rationale of this investigation was to systematically review scientific literature with at least a decade of follow-up comparing the ISR of implants placed in grafted and non-grafted sites.

The aim was to systematically review clinical studies which, compared the ISR after at least 10 years for dental implants placed in grafted and non-grafted sites.

## MATERIALS AND METHODS

### Protocol, Focused Question and PICO

The present systematic review was performed based on the latest Preferred Reporting Items for Systematic Reviews and Meta-Analyses (PRISMA) guidelines.^29^ The Patients, Intervention, Control and Outcome (PICO) (P = patients with dental implants in function for at least a decade; I = implants placed in grafted sites; C = Implants placed in non-grafted sites; O = implant survival rate [ISR]) format was used to address the following focused question: ‘Is there a difference in the ISR of dental implants placed in grafted and non-grafted sites for at least a decade?’.

### Inclusion and Exclusion Criteria

The inclusion criteria were as follows: (a) longitudinal controlled clinical studies with at least 10 years of follow-up; (b) adult patients (over the age of 18 years) undergoing dental implant therapy; (c) implant placement in grafted sites (maxilla and/or mandible); (d) implants placed without GBR; (e) evaluation of clinical (including but not limited to plaque index [PI], gingival index [GI]/ bleeding on probing [BOP], and probing depth [PD]) and radiographic status (CBL); (f) ISR; and (g) statistical analysis. Case series, case reports, letters to the editor, review articles, commentaries, *in-vitro*/*ex-vivo*/*in-vivo* studies, and studies on animal models were excluded.

### Information Sources, Search Strategy and Study Selection

An electronic search was conducted in indexed databases (PubMed [National Library of Medicine], EMBASE, Scopus, ISI Web of Knowledge and Google Scholar) without language and time restrictions from inception up to and including May 2024. The following medical subject headings were used: (a) dental implant; (b) bone regeneration; (c) bone grafting; (d) 10 years; (e) survival. These keywords were combined using Boolean operators (OR, AND) to expand the search results. The author (SG) screened the titles and abstracts of studies identified with the above-mentioned protocol, and full texts of relevant studies were read independently. Hand-searching of the reference lists of pertinent original studies and review articles was also performed to identify studies that might have been missed in the previous step. A second researcher was consulted in case there were doubts regarding the eligibility of a study in terms of its inclusion or exclusion.

### Data Collection and Data Items

Data extraction from eligible studies was independently performed by the author (SG); and the following information was collected: (a) reference; (b) study design; (c) number of participants and study groups; (d) demographic information; (e) information regarding GBR; (f) information regarding implant loading; (g) duration of implants in function; (h) investigative parameters/clinicoradiographic parameters; (i) main outcomes (ISR); (j) conclusion; and (k) quality assessment. Any doubts related to the parameters were resolved via discussion with a colleague researcher.

### Risk of Bias and Level of Evidence Assessment

The risk of bias was assessed using the Risk of Bias in Non-randomised Studies of Interventions (ROBINS-I) tool.^40^ The Oxford Centre for Evidence-Based Medicine (OCEBM) classification^20^ was used to evaluate the reliability and validity of the evidence supporting clinical and radiographic outcomes of dental implants placed in grafted and non-grafted sites over a decade of follow-up. Based upon the study design, the studies were categorised as level 1 (high-quality evidence), level 2 (moderate quality evidence), level 3 (low-quality evidence), level 4 (very low-quality evidence) and level 5 (expert opinion).^20^

## RESULTS

### Overview of Search Strategy

**Fig 1:**
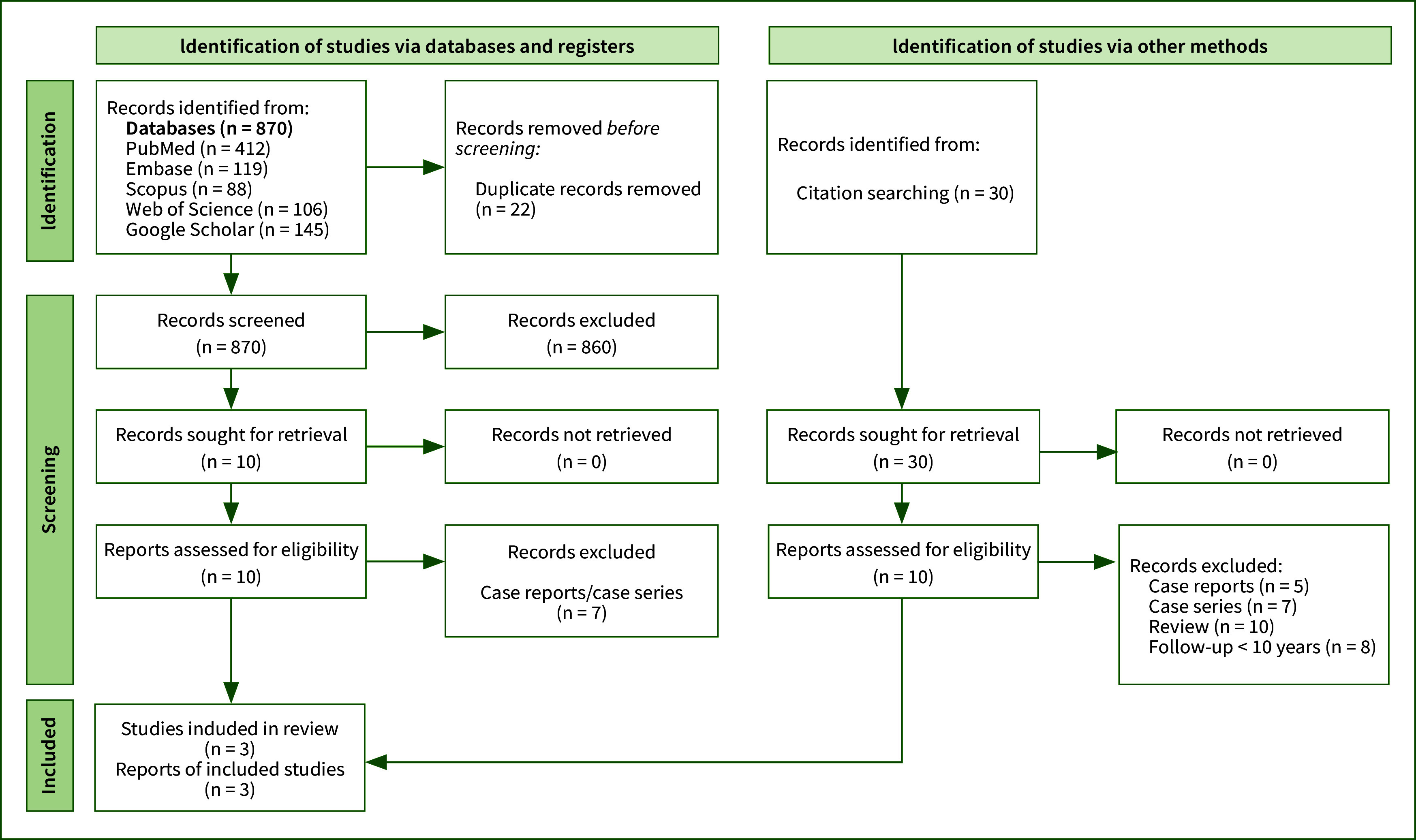
Identification of studies via databases according to PRISMA guidelines.

The initial search yielded 412 studies through electronic database searching. An additional 30 studies were identified through manual searching of full texts of studies. A total of 420 studies that did not address the focused question and/or were duplicates were excluded. The remaining 10 studies were reassessed, and six case reports/case series were further excluded. In total, three studies^9,10,24^ were included and processed for data extraction (Fig 1).

### General Characteristics of the Studies Assessed

The study by Roccuzzo et al^24^ was a non-randomised clinical study, whereas studies by Daubert et al^9^ and de Moraes et al^10^ had a cross-sectional study and retrospective cohort study design, respectively. In these studies, the number of participants ranged between 34 and 96 individuals. The mean age of participants ranged between 47.2 and 67.6 years, respectively. There was no statistically significant difference in the distribution of males and females in all studies.^9,10,24^ In all studies, the participants were partially edentulous (Table 1). According to OCEBM levels of classification,^20^ one study was judged to be level 3,^24^ whereas studies by Daubert et al^9^ and De Moraes et al^10^ were classified as level 4 studies.

**Table 1:** General characteristics of the included clinical studies

Authors	Study design	Participants	Age in years	Gender	Dental status	Level of evidence
**Daubert et al** ^9^	**Cross-sectional case-control study**	**59 patients with GBR** **37 patients without GBR**	**67.6 years**	**50% males** **50% females**	**Partially edentulous**	**4**
**De Moraes et al** ^10^	**Retrospective case-control cohort study**	**22 patients with Autogenous bone graft** **20 patients with no graft**	**59.3 years** **60.2 years**	**69.2% males** **30.8% females** **66.6% males** **33.4% females**	**Edentulous**	**4**
**Roccuzzo et al** ^24^	**Non-randomised case-control study**	**19 patients with GBR** **15 patients without GBR**	**48.4 years** **47.2 years**	**62.2% males** **37.8% females** **53% males** **43% females**	**Partially edentulous**	**3**
**EL = early loading; GBR = guided bone regeneration; IL = immediate loading**	

### Dental Implant-related Parameters

Thirty-four implants manufactured by Straumann (Basel, Switzerland) were used in the study by Roccuzzo et al^24^; whereas Daubert et al^9^ used 225 implants (11 immediately and 85 delayed loaded) from various manufacturers. The implant loading protocol (immediate, early, or delayed) was not reported in the study by Roccuzzo et al.^24^ In the study by de Moraes et al,^10^ 306 implants were used. In all studies,^9,10,24^ the implants were in function for 10 years. In the studies by Roccuzzo et al^24^ and Daubert et al,^9^ the implant survival rates were 100% and 91.6%, respectively. In the study by de Moraes et al,^10^ the ISR was 96% in the graft group and 94% in the non-grafted group (Table 2).

**Table 2:** Implant-related characteristics of the included clinical studies

Authors	Total implant assessed	Implant loading protocol	Dental prosthesis	Duration of implants in function	Type of grafting	Implant survival rate
**Daubert et al** ^9^	**225 implants**	**Immediate loading (n = 11 implants)** **Delayed loading (n = 85 implants)**	**Fixed single unit**	**10 years**	**NR**	**Implants with GBR: 100%** **Implants without GBR: 91.6%**
**De Moraes et al** ^10^	**306 implants**	**Delayed loading**	**Fixed single unit**	**10 years**	**Bone reconstruction**	**Implants with GBR: 96%** **Implants without GBR: 94%**
**Roccuzzo et al** ^24^	**34 implants**	**NR**	**Fixed single unit**	**10 years**	**Alveolar ridge preservation**	**Implants with GBR: 100%** **Implants without GBR: 100%**


### Confounding Factors

In the study by de Moraes et al,^10^ patients with a history of tobacco smoking were excluded; and in the remaining,^9,24^ this parameter remained unclear. All studies^9,10,24^ did not report whether patients with a history of periodontitis were excluded. In the study by Roccuzzo et al,^24^ patients with self-reported systemic diseases were excluded, and in two studies,^9,10^ it remained unclear whether or not patients with self-reported systemic diseases were excluded. A prior sample-size estimation (power analysis) was performed in none of the studies^9,10,24^ (Table 3).

**Table 3:** Confounding factors in the studies assessed

Authors	Were smokers excluded?	Were periodontitis patients excluded?	Were patients with systemic diseases excluded?	Was power analysis done?
**Daubert et al** ^9^	**No**	**No**	**No**	**No**
**De Moraes et al** ^10^	**Yes**	**No**	**No**	**No**
**Roccuzzo et al** ^24^	**No**	**Yes**	**Yes**	**No**


### Risk of Bias Assessment

**Fig 2:**
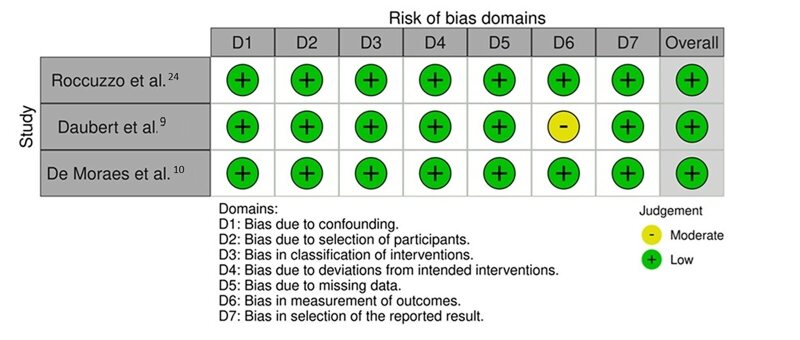
ROBINS-I tool to assess risk of bias in non-randomised clinical studies.

All the included studies^9,10,24^ were judged to be low risk of bias (RoB), as shown in Figure 2.

## DISCUSSION

During the initial literature search, the authors identified case reports and case series that assessed the survival of dental implants placed in grafted sites at 10 years of follow-up; however, three clinical investigations^9,10,24^ addressed the focused question and processed for data extraction.

Although case reports and case series greatly influence literature related to health sciences and help advance our knowledge; such studies (case reports and case series) are based on the presentation and follow-up of one patient or a limited number of patients. Moreover, in case reports and case series, there is no control group, that is, patients who did not receive the intervention under investigation (bone grafting as in the current scenario). In this context, there is a potentially high RoB among case reports and case series. Keeping such limitations in mind, the author of the present systematic review excluded case reports and case series during the literature search.

The results of studies^9,10,24^ evaluated in the present systematic review showed that all implants placed in grafted and non-grafted sites had an ISR ranging between approximately 90% and 100% at 10 years of follow-up as depicted in Table 2. From this outcome, it is tempting to speculate that dental implants osseointegrate and remain functionally stable in grafted as well as non-grafted sites with no significant difference among the former and latter groups. Conversely, such a statement should be cautiously interpreted as some critical methodology-based factors may potentially have biased the results. It is noteworthy that there was a discrepancy in the methodology of all studies assessed. For instance, it remained unclear whether tobacco smokers were excluded in 75% of the studies^9,10,24^ assessed. Smoking is a classical risk factor for crestal bone loss around dental implants.^33–36^ Tobacco smoke is known to contain chemicals, of which nicotine is the most harmful.^14^ At a cellular level, nicotine reduces the proliferation of erythrocytes, macrophages and fibroblasts and enhances the adhesiveness of platelets.^38^ In addition, nicotine compromises healing and tissue repair due to micro-clot formation in the blood vessels.^27,38^ Nicotine exerts a sympathomimetic action, thereby causing vasoconstriction and limiting tissue perfusion.^21^ Considering these effects, it is likely that nicotine might impair healing potential at the bone/implant interface. Yamano et al^45^ reported that nicotine downregulates the expression of bone matrix-related genes and a decrease in bone formation around implants placed in rats exposed to nicotine.

On a similar note, Berley et al^6^ reported a decreased bone-to-implant contact following implant placement in rats exposed to nicotine compared with control rats that received saline injections. From a clinical point of view, detrimental effects of smoking on implant survival have been reported. In a recent study, Mumcu and Beklen^28^ showed that smoking augments bone loss around implants and jeopardises their success and survival.^28^ Furthermore, smoking also compromises the outcome of oral surgical procedures, including dental implant therapy.^17^ Likewise, immunosuppression, which often manifests in patients with poorly controlled diabetes mellitus, is another factor that compromises osseous healing and jeopardises ISR.^18,19^

It seems that patients with a compromised systemic health status were excluded from the studies^9,10,24^ included in the present systematic review. It is speculated that failure of graft and osseointegration is more often manifested around implants placed in grafted and non-grafted sites, respectively. However, further prospective studies are needed to test this hypothesis. The author also noticed a discrepancy in the implant loading protocol among the studies assessed. For instance, in the two studies,^9,24^ implant loading protocol was not reported; and in the study by de Moraes et al,^10^ delayed loading protocol was used. To the author’s knowledge from pertinent indexed literature, the impact of implant loading on implant survival in grafted and non-grafted sites remains unclear. In this regard, further studies are warranted. Furthermore, the grafting protocol remained poorly described in the studies included^9,10,24^ in the present systematic review. None of the studies reported the precise surgical protocol (quantity of bone graft used, use of membranes, experience of the operator), and the use of adjuvant treatments during osseous grafting remained dubious.

Finally, none of the studies^9,10,24^ had a power-adjusted patient population. In clinical and experimental studies, power analysis for sample-size estimation,^4^ and blinding of the outcome assessors^5,31^ are essential factors that tend to minimise the RoB. These are critical factors that seem to have biased the results of the studies that fulfilled the eligibility criteria.^9,10,24^ Based on the results of the present study, it is recommended that oral healthcare providers should focus on comprehensive patient education, stressing the critical role of meticulous oral hygiene maintenance and deleterious effects of habits such as use of nicotinic products and habitual alcohol intake on oral and implant health. Moreover, patients should be instructed on proper cleaning techniques and the use of adjunctive aids such as flossing of interproximal spaces between teeth and implants. Furthermore, routine dental checkups and prophylaxis are essential for early detection and management of any oral and peri-implant complications. Such measures are anticipated to contribute towards the long-term success and survival of dental implants placed in grafted and non-grafted sites. However, further longitudinal and well-designed studies are needed in this regard.

## CONCLUSION

The long-term (10-year) survival rates of implants placed in grafted and non-grafted sites are similar. However, further well-designed and power-adjusted studies are needed.
